# Radiation-Activated PI3K/AKT Pathway Promotes the Induction of Cancer Stem-Like Cells via the Upregulation of SOX2 in Colorectal Cancer

**DOI:** 10.3390/cells10010135

**Published:** 2021-01-12

**Authors:** Ji-Hye Park, Young-Heon Kim, Sehwan Shim, Areumnuri Kim, Hyosun Jang, Su-Jae Lee, Sunhoo Park, Songwon Seo, Won Il Jang, Seung Bum Lee, Min-Jung Kim

**Affiliations:** 1 Laboratory of Radiation Exposure & Therapeutics, National Radiation Emergency Medical Center, Korea Institute of Radiological and Medical Sciences, Seoul 01812, Korea; parkjh0420@kirams.re.kr (J.-H.P.); bumbroberto1@nate.com (Y.-H.K.); ssh3002@kirams.re.kr (S.S.); nurikim@kirams.re.kr (A.K.); hsjang@kirams.re.kr (H.J.); sunhoo@kirams.re.kr (S.P.); zzangIl@kirams.re.kr (W.I.J.); 2 Department of Life Science, Research Institute for Natural Sciences, Hanyang University, Seoul 04763, Korea; sj0420@hanyang.ac.kr; 3 Department of Pathology, Korea Cancer Center Hospital, Korea Institute of Radiological and Medical Sciences, Seoul 01812, Korea; 4 Laboratory of Radiation Epidermiology, National Radiation Emergency Medical Center, Korea Institute of Radiological and Medical Sciences, Seoul 01812, Korea; seo@kirams.re.kr

**Keywords:** colorectal cancer, cancer-stem like cells, radioresistance, SOX2, PI3K/AKT

## Abstract

The current treatment strategy for patients with aggressive colorectal cancer has been hampered by resistance to radiotherapy and chemotherapy due to the existence of cancer stem-like cells (CSCs). Recent studies have shown that SOX2 expression plays an important role in the maintenance of CSC properties in colorectal cancer. In this study, we investigated the induction and regulatory role of SOX2 following the irradiation of radioresistant and radiosensitive colorectal cancer cells. We used FACS and western blotting to analyze SOX2 expression in cells. Among the markers of colorectal CSCs, the expression of CD44 increased upon irradiation in radioresistant cells. Further analysis revealed the retention of CSC properties with an upregulation of SOX2 as shown by enhanced resistance to radiation and metastatic potential in vitro. Interestingly, both the knockdown and overexpression of SOX2 led to increase in CD44+ population and induction of CSC properties in colorectal cancer following irradiation. Furthermore, selective genetic and pharmacological inhibition of the PI3K/AKT pathway, but not the MAPK pathway, attenuated SOX2-dependent CD44 expression and metastatic potential upon irradiation in vitro. Our findings suggested that SOX2 regulated by radiation-induced activation of PI3K/AKT pathway contributes to the induction of colorectal CSCs, thereby highlighting its potential as a therapeutic target.

## 1. Introduction

Colorectal cancer is one of the most common malignancies and the fourth leading cause of cancer death in the world. The current treatment strategy for patients with aggressive colorectal cancer has been hampered by their resistance to radiotherapy and chemotherapy [1,2]. Growing evidence indicates that the existence of a small population of cancer cells known as cancer stem-like cells (CSCs) is responsible for tumor recurrence and is the main cause of treatment resistance in many cancers, including glioma, breast, oral, and colorectal cancer. CSCs further exhibit diverse cancer-initiating properties such as self-renewal and metastatic potential [3,4,5,6,7]. For the identification of CSCs, several putative markers such as transmembrane glycoprotein (CD133) and the cell-surface glycoprotein (CD44) were reported to be expressed in colorectal cancer and correlated with high-risk cases and a poorer survival rate of colorectal cancer patients [8,9]. However, the molecular subclassification of CSCs based on their cancer-promotion property in colorectal cancer needs to be understood.

The stemness program is involved in maintaining the properties of CSCs, such as self-renewal and cancer-initiation, which are the hallmarks of cancer cells. SOX2 is a member of the SRY-related HMG-box (SOX) gene family and play an essential role in the maintenance of a pluripotent state in stem cells and cell-fate determination during developmental processes [10,11]. SOX2 is one of the key molecules driving CSC properties. SOX2-expressing cancer cells, such as those in skin and bladder carcinomas, express high levels of CSC markers, depending on tissue origin, and reveal enhanced tumorigenicity [12,13]. Recent studies have shown that SOX2 is aberrantly expressed and involved in the maintenance of CSCs. The properties of CSCs, including spheroid-like growth and metastatic potential, were observed in SOX2-positive colorectal cancer with an increased expression of CSC markers such as CD44 [14,15]. Moreover, Ghisolfi et al. recently showed that environmental stresses such as radiation induced the expression of both mRNA and protein of SOX2 and regulated CSCs in hepatocellular carcinoma cells [16]. However, the induction and the signaling pathways of SOX2 upon environmental stress in colorectal CSCs remain unclear.

In this study, we investigated the induction and regulatory role of SOX2 in colorectal CSCs following radiation exposure in both radioresistant and radiosensitive colorectal cancer cell lines.

## 2. Materials and Methods

### 2.1. Antibodies and Reagents

Anti-CD44, anti-phospho-AKT, anti-AKT, anti-phospho-ERK, anti-ERK1/2, anti-phospho-p38, anti-p38, anti-phospho-SAPK/JNK, and anti-JNK1/2 antibodies were purchased from Cell Signaling Technology (Cambridge, MA, USA). Anti-phospho-JNK, anti-Oct3/4 and anti-β-actin were obtained from Santa Cruz Biotechnology (Santa Cruz, CA, USA). Anti-SOX2, anti-Notch2, and anti-Snail were purchased from Abcam (Cambridge, MA, USA). Anti-β-catenin was procured from BD Bioscience (Franklin Lakes, NJ, USA). For transfection, non-targeting siRNA and commercial siRNA for SOX2 or Snail or AKT were purchased from Genolution (Genolution Pharmaceuticals, Seoul, Korea). The cells were transfected with each siRNA (50 nM) for 48 h using Lipofectamine 2000 (Invitrogen, Carlsbad, CA, USA), as described in the manufacturer’s procedure.

### 2.2. Cell Culture and Irradiation

HT29, DLD1, HCT116, SW480, RKO, and LoVo colorectal cancer cells were purchased from the Korean Cell Line Bank (KCLB, Seoul, Korea). These cell lines from passages 4 to 10 were used for the experiments and were maintained in RPMI 1640 medium containing 1% antibiotic-antimycotic (GIBCO) supplemented with 10% FBS. The cells were cultured in a humidified 5% CO_2_ atmosphere at 37 °C. Cells were exposed to radiation (0~10 Gy) using a Gammacell 3000 Elan irradiator (137Cs γ-ray source; MDS Nordion, Canada).

### 2.3. Flow Cytometry and Aldehyde Dehydrogenase (ALDH) Assay

Irradiated cells were gently dissociated and incubated with anti-CD44-Fluorescein (FITC) or anti-CD133-phycoerythrin (PE) antibody (eBioscience Inc., San Diego, CA, USA) for 30 min on ice. The samples were washed twice with PBS containing 0.1% BSA and EDTA, and the cells were resuspended in PBS containing 0.1% BSA and EDTA and analyzed using FACSCalibur and CellQuest programs (BD Biosciences, San Jose, CA, USA). For ALDH assay, an ALDELUOR kit (Stemcell Technologies Inc., Vancouver, BC, Canada) was used to detect the cell population with high ALDH enzymatic activity, according to the manufacturer’s procedure, after which the cells were analyzed by flow cytometry. Apoptotic cells were analyzed as previously described [17].

### 2.4. Fluorescence-Activated Cell Sorting (FACS Sorting)

To isolate CSCs from colorectal cancer cell lines, the cells were gently dissociated and incubated with anti-CD44-FITC antibody for 30 min on ice. CD44+ positive cells were sorted by a using a FACS Vantage SE flow cytometer (BD Biosciences) equipped with FlowJo software (Tree Star, Ashland, OR, USA).

### 2.5. Immunocytochemistry (ICC)

The cells were fixed with 4% paraformaldehyde and permeabilized with 0.1% Triton X-100 in PBS. Following fixation, cells were incubated at 4 °C overnight with anti–CD44 or anti–SOX2 primary antibodies in PBS with 1% BSA and 0.1% Triton X-100. Stained proteins were visualized using secondary antibodies against anti-mouse immunoglobulin/FITC or anti-rabbit immunoglobulin/PE (1:400, BD Bioscience). Nuclei were counterstained with DAPI (Sigma, St. Louis, MO, USA). Following staining, cells were observed with an Olympus IX71 fluorescence microscope (Olympus, Tokyo, Japan).

### 2.6. Western Blot Analysis

The cells were lysed using lysis buffer (40 mM Tris-HCl [pH 8.0], 120 mM NaCl, 0.1% Nonidet-P40) supplemented with protease and phosphatase inhibitors. The protein concentration was measured using the Bradford assay (Bio-rad, Hercules, CA, USA). Equal amounts of total protein in cell lysates were separated by 10% SDS-PAGE and transferred to a nitrocellulose membrane (Amersham, UK). The membranes were blocked with 5% skim milk for 1 h at room temperature, incubated with primary antibodies (1:1000) overnight at 4 °C. Blots were developed with the appropriate horseradish peroxidase (HRP)-conjugated secondary antibody (anti-mouse IgG-HRP or anti-rabbit IgG-HRP; Cell Signaling Technology, Danvers, MA, USA), and proteins were visualized using enhanced chemiluminescence (ECL) procedures.

### 2.7. Apoptosis Assay

Apoptosis analysis was performed using a FITC Annexin V Apoptosis Detection Kit I (BD Biosciences, Waltham, MA, USA), according to the manufacturer’s instructions. Briefly, radiation-induced apoptotic cells were collected at the indicated time points and resuspended in 1× diluted binding buffer in Kit. For staining, Annexin V-FITC and PI were added to each sample, and the mixture was incubated for 5 min at room temperature in the dark. The cells were analyzed immediately using a BD FACS CANTO II flow cytometer (BD Biosciences).

### 2.8. Colony Formation Assay

Colony formation assay was performed as previously described [17]. To test the effect of IR on cell viability, appropriately seeded cells were irradiated with different doses of radiation (0, 0.5, 1, 2, 3, or 4 Gy) and incubated continuously for 2 weeks. The colonies were stained with 1% crystal violet. Colonies containing >50 cells were scored as surviving cells.

### 2.9. Invasion and Migration Assays

Invasion assay was performed as previously reported [18]. Briefly, the cells treated with either inhibitors or siRNAs for transfection were seeded in the upper well of a Transwell chamber (8-μm pore size) that was pre–coated with 10 mg/mL growth factor-reduced Matrigel (BD Bioscience). After 72 h, non–invading cells on the upper surface of the filter were removed with a cotton swab, and the migrated cells on the lower surface of the filter were fixed and stained with a Kwik-Diff kit (Thermo Fisher Scientific, Waltham, MA, USA). Invasiveness was determined by counting cells in fields per well, and the extent of invasion was expressed as the average number of cells per microscopic field. The cells were imaged by phase contrast microscopy. For the migration assay, we used Transwell chambers with inserts that contained the same type of membrane but without the Matrigel coating.

### 2.10. Tumoursphere-Formation Assay

After producing a single-cell suspension, the cells were cultured in ultra-low attachment 6-well or 96-well plates in medium consisting of DMEM/F12 supplemented with 2% B27 supplements (Invitrogen), 10 ng mL^−1^ bFGF (Peprotech, Rocky Hill, NJ, USA) and 10 ng/mL EGF. The cells were cultured for 7 days, and the morphology and size of sphere were determined using an Olympus IX71 fluorescence microscope (Olympus).

### 2.11. Statistical Analysis

Statistical significance of the differences between mean values was calculated with pairwise comparisons using Least Significance Difference (LSD) test after a one-way Analysis of Variance (ANOVA). Due to the exploratory nature of this study, multiplicity adjustment was not made. Statistical analyses were conducted using SPSS (version 12.0; SPSS Inc., Chicago, IL, USA) or Excel (Microsoft, Redmond, WA, USA) software packages.

## 3. Results

### 3.1. Radiation Increased the Population of Radioresistant Rather Than Radiosensitive CD44+ Colorectal Cancer Cells

**Figure 1:**
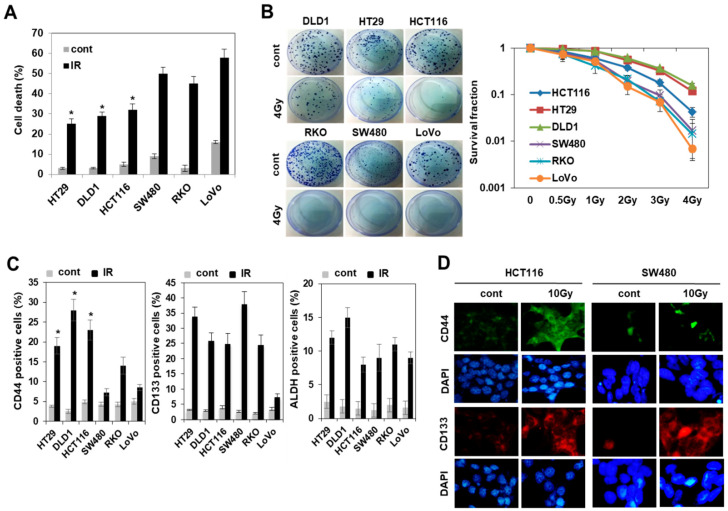
Enriched population of CD44 positive (CD44+) cells was found in radioresistant cells and not in radiosensitive cells of colorectal cancer in response to radiation. (**A**) The apoptotic cells on day 2 after irradiation with 10 Gy were measured by annexin V staining and flow cytometry analysis in various types of colorectal cancer cell lines including both radioresistant cells (HT29, DLD1, and HCT116) and radiosensitive cells (SW480, RKO, and LoVo). Data are shown as mean ± SD (*n* = 3) with * *p* < 0.05 for the pairwise comparisons between radioresistant cells and radiosensitive cells. (**B**) Colony formation assay was performed with indicated cells treated with 4 Gy (left panel). Graph showing quantification of relative colony numbers at different doses of IR (right panel). (**C**) Cell populations for the CD44+, CD133+, or ALDH+, which are known markers of cancer stem-like cells (CSC) in these indicated cells after radiation exposure were measured by flow cytometric analysis. The percentage of each CSC marker-expressing cell is shown as a bar graph. Data are shown as mean ± SD (*n* = 3) with * *p* < 0.05 for the pairwise comparisons between radioresistant cells and radiosensitive cells. (**D**) Cells were stained with an anti-CD44 antibody (green) and anti-CD133 antibody (red). Nuclei were counterstained with DAPI ((blue). CSCs: cancer stem-like cells.

Experimental and clinical data show that CSCs play an important role in tumor recurrence and resistance to therapy [7,8,9]. To investigate the relationship between radiation resistance and population of CSCs, we first confirmed the radiation resistance in various types of colorectal cancer cells, including in previously reported radioresistant and radiosensitive cells [17]. Colorectal cancer cells such as HCT116, DLD1, and HT29 were relatively resistant to radiation by annexin V/PI staining and colony formation assay. (Figure 1A,B); however, the expression of colorectal CSC markers such as CD44, CD133, and ALDH was similar to that of radiosensitive colorectal cancer cells under untreated conditions (Figure 1C). Next, we examined the effect of radiation on the expression of colorectal CSC markers. Flow cytometry and immunocytochemistry analysis (Figure 1C,D) showed that radiation increased the expression of CD133 and ALDH in all colorectal cancer cells except LoVo cells with unchanged expression of CD133, whereas CD44 expression was selectively increased in radioresistant colorectal cancer cells such as HCT116, DLD1, and HT29 (Figure 1C). These results suggested that resistance to radiation in radioresistant colorectal cancer cells may be acquired by radiation-upregulated expression of CD44, which is one of the markers of colorectal CSCs.

### 3.2. Radiation-Enriched CD44+ Cells Exhibited the Properties of CSCs Including an Increase in SOX2 Expression

**Figure 2:**
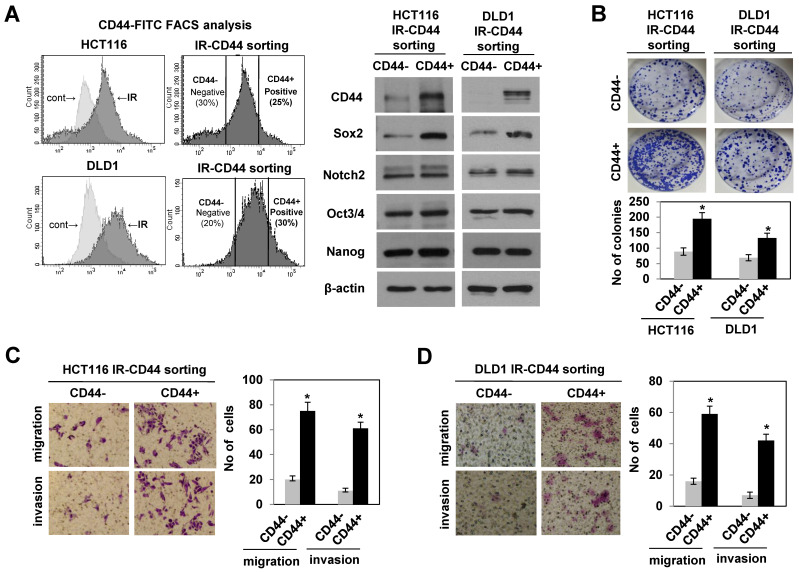
CD44+ cells induced by radiation exhibited the properties of cancer stem-like cells (CSCs) with an increase in SOX2 levels. (**A**) CD44+ CD44− cells on day 2 after irradiation with 10 Gy in radioresistant colorectal cancer cells (HCT116 and DLD1) were sorted (left panel). Immunoblotting for the expression of CSC-related proteins in CD44+ (positive) and CD44− (negative) in radioresistant cells (right panel). (**B**) Colony formation assay was performed with CD44+ (or CD44−) cells, and the bar graphs show the quantification of relative colony numbers in indicated cells. Data are shown as mean ± SD (*n* = 3) * *p* < 0.05 compared to control. (**C**,**D**) The migration and invasion analysis (left panel) and quantification of cells involved in migration and invasion (right panel) in CD44+ and CD44− cells sorted from HCT116 and DLD1 cells, respectively. All experiments were performed in triplicates. Data are shown as mean ± SD. * *p* < 0.05 compared to CD44− cell. CD44−: negative, CD44+: positive, CSCs: cancer stem-like cells.

To delineate the role of radiation-induced CD44 expression in radioresistant colorectal cancer cells, we isolated both CD44 positive (CD44+) and negative (CD44−) cells in HCT116 and DLD1 cells following irradiation using anti-CD44-FITC antibodies by FACS, and the expression of CD44 in both CD44+ and CD44− cells is shown in Figure 2A. Since the CD44 marker correlated with the features of CSCs in colorectal cancers [19,20], we evaluated the properties of colorectal CSCs including metastatic potential and self-renewal. We observed an increase in colony formation, migration and invasion in the sorted CD44+ cells after irradiation and not in CD44− cells in both cell lines (Figure 2B–D). Interestingly, immunoblotting of stemness-related proteins revealed significant elevation in SOX2 levels among stemness-related proteins [21,22] on sorted CD44+ cells (Figure 2A). Given the evidence that SOX2 was aberrantly expressed and involved in the maintenance of CSCs in colorectal cancer [14,15], these results indicated the possibility of a functional relationship between SOX2 expression and CD44-mediated CSC property in radioresistant cells upon radiation exposure.

### 3.3. Modulation of SOX2 Expression in Colorectal Cancer Cells Is Associated with Induction of Colorectal CSCs Following Irradiation

**Figure 3:**
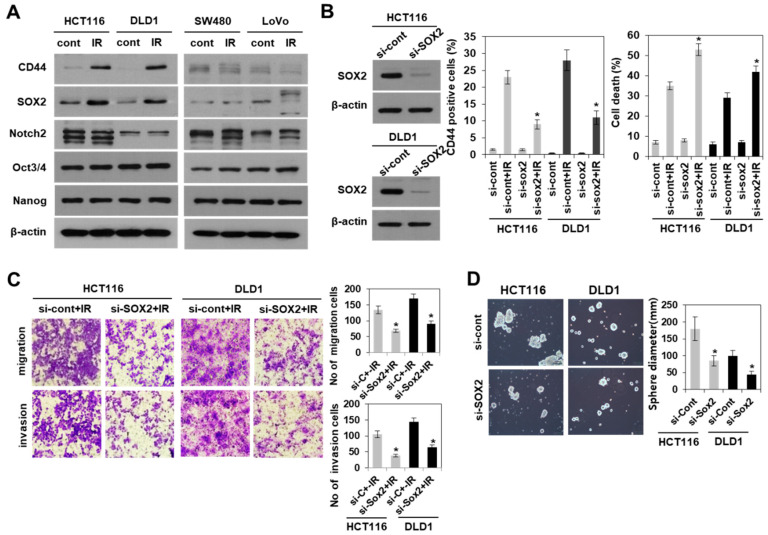
Knockdown of SOX2 in radioresistant colorectal cancer cells attenuated the induction of colorectal CSCs after irradiation. (**A**) Immunoblotting for the expression of CSC-related proteins on day 2 after radiation (10 Gy) in colorectal cancer cells as indicated. (**B**) siRNA-mediated SOX2 knockdown in cells was identified by western blotting (left) and CD44+ cell population (middle), or apoptotic cells were analyzed by flow cytometry (right). All experiments were performed with the SOX2 siRNA-transfected HCT116 and DLD1 cells on day 2 after radiation (10 Gy). Data are shown as mean ± SD (*n* = 3). * *p* < 0.05 compared si-Cont + IR to si-SOX2 + IR. (**C**) The images of migration and invasion on day 2 after radiation (10 Gy) of the SOX2 siRNA-transfected HCT116 and DLD1 cells were quantified. Bars indicate measurements of migration and invasion. * *p* < 0.05 compared si-Cont + IR to si-SOX2 + IR. (**D**) Tumoursphere-formation assay was performed to evaluate self-renewal ability of CSCs in SOX2 siRNA-transfected cells. Indicated cells were seeded in a non-adherent culture condition. After culturing for 7 days, the number of tumoursphere cells (>100 μm diameter) was quantified. Data are shown as mean ± SD (*n* = 3). * *p* < 0.05 versus si-Cont. IR: irradiation, Si-Cont: control siRNA, si-SOX2: SOX2 siRNA, CSCs: cancer stem-like cells.

**Figure 4:**
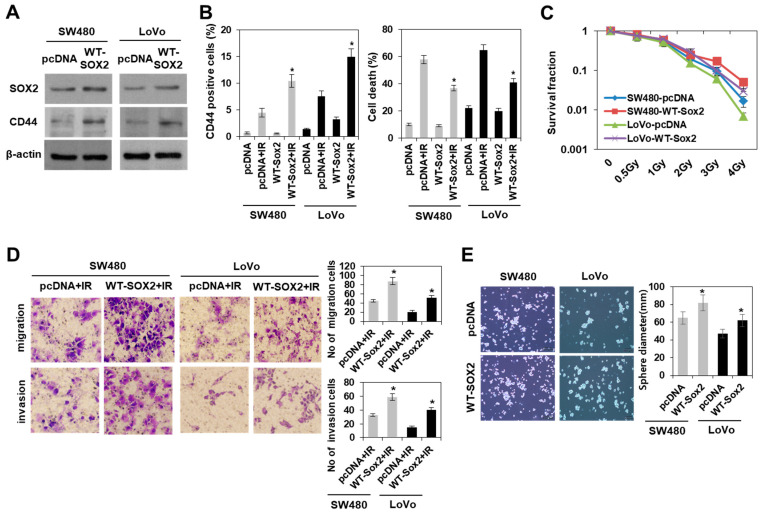
SOX2 overexpression in radiosensitive colorectal cancer cells facilitated the induction of colorectal CSCs following irradiation. (**A**) Immunoblotting of SOX2 and CD44 in SOX2-overexpressing radiosensitive colorectal cancer cells (SW480 and LoVo) on day 2 after irradiation with 10 Gy. (**B**) Analysis of CD44+ cell population (left panel) and apoptotic cells (right panel) by flow cytometry in SOX2-overexpressing SW480 and LoVo cells. Data are shown as mean ± SD (*n* = 3). * *p* < 0.05 compared pcDNA + IR to WT-SOX2 + IR. (**C**) Colony formation assay was performed with SOX2-overexpressing SW480 and LoVo cells, and graph showing the quantification of relative colony numbers at different doses of radiation. Data are shown as mean ± SD (*n* = 3). (**D**) The images of migration and invasion on day 2 after radiation (10 Gy) of SOX2-overexpressing SW480 and LoVo cells were quantified. Bars indicate the measurements of migration and invasion. * *p* < 0.05 compared pcDNA + IR to WT-SOX2 + IR. (**E**) Tumoursphere-formation assay was performed to evaluate self-renewal of CSCs in SOX2-overexpressing SW480 and LoVo cells. Indicated cells were seeded in a non-adherent culture condition. After culturing for 7 days, the images and size of tumoursphere cells were analyzed. Data are shown as mean ± SD (*n* = 3). * *p* < 0.05 versus Cont.

We further determined whether the expression of either CD44 or SOX2 in response to radiation is dependent on radioresistant colorectal cancer cells. These proteins were upregulated by irradiation in radioresistant, but not radiosensitive colorectal cancer cells (Figure 3A). To further clarify the role of radiation induced SOX2 in regulating colorectal CSCs, we examined the effect of SOX2 siRNA on the properties of CSCs in both HCT116 and DLD1 cells. Immunoblotting analysis in Figure 3B showed the efficient knockdown of SOX2 expression in both cells with SOX2 siRNA treatment. In addition, the knockdown of SOX2 attenuated the radiation-induced properties of CSCs, including the enhanced ability to migrate, invade, and form tumourspheres, and reduced CD44+ population growth (Figure 3B–D). Next, we examined the effects of SOX2 overexpression. Upon irradiation, the overexpression of SOX2 facilitated the acquisition of the properties of colorectal CSCs in radiosensitive colorectal cancer cells (SW480 and LoVo) due to an increase in CD44+ population, cell survival, migration, invasion, and tumoursphere-formation (Figure 4). Taken together, these results suggested that SOX2 regulated population growth and properties of CSCs in colorectal cancer following irradiation, and SOX2 may be a potential target for studies involving resistance to radiation.

### 3.4. Radiation-Induced Activation of the PI3K/AKT Pathway, but Not the MAPK Pathway Modulated SOX2-Dependent Induction of Colorectal CSCs

**Figure 5:**
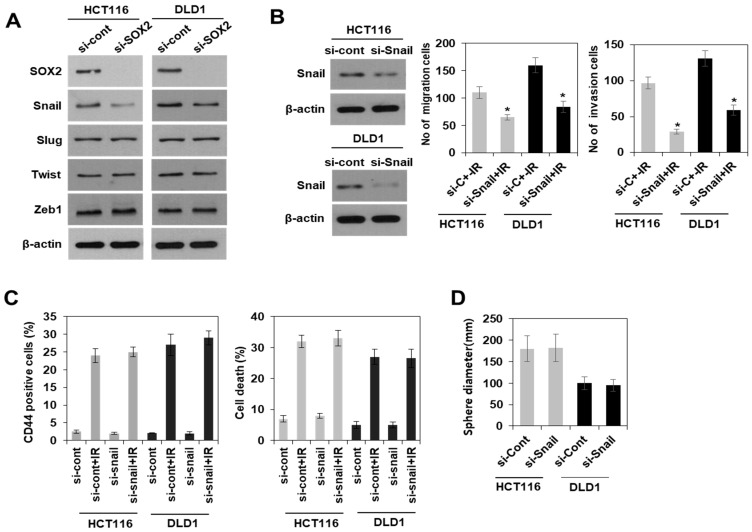
SOX2-dependent induction of colorectal CSCs was not associated with Snail-promoted ability of migration and invasion after irradiation. (**A**) Immunoblotting for SOX2 and EMT regulator (Snail, Slug, Twist, and Zeb1) in SOX2 siRNA-transfected radioresistant colorectal cancer cells (HCT116 and DLD1). (**B**) siRNA-mediated Snail knockdown cells were identified by western blotting (left) and the migration and invasion (right) on day 2 after radiation (10 Gy), of the Snail siRNA-transfected HCT116 and DLD1 cells were quantified. Bars indicate measurements of migration and invasion. * *p* < 0.05 compared si-Cont + IR to si-Snail + IR. (**C**) Analysis of CD44+ cell population (left panel) and apoptotic cells (right panel) by flow cytometry from irradiated cells with 10 Gy on day 2. Data are shown as mean ± SD (*n* = 3). (**D**) Tumoursphere-formation assay was performed to evaluate self-renewal of CSCs in Snail siRNA-transfected cells. Indicated cells were seeded in a non-adherent culture condition. After culturing for 7 days, the size of tumoursphere cells was measured. Data are shown as mean ± SD (*n* = 3). EMT: epithelial-mesenchymal transition, si-Cont: control siRNA, si-Snail: Snail siRNA, CSCs: cancer stem-like cells.

Next, the potential molecular mechanism involved in the SOX2-dependent induction of colorectal CSCs following irradiation was elucidated. The phenomenon of epithelial-mesenchymal transition (EMT) has emerged as a feature of CSCs in recent times [6,23]. In addition, expression levels of the master regulator of EMT such as Snail and Zeb1/2 were modulated by SOX2 protein level [24,25]. Therefore, we investigated whether EMT is associated with SOX2-dependent induction of colorectal CSCs. Immunoblotting analysis showed that among EMT-associated proteins, Snail expression was decreased in SOX2 siRNA-transfected HCT116 and DLD1 cells (Figure 5A). In addition, we found that knockdown of Snail dramatically suppressed the ability of migration and invasion, a hallmark of EMT (Figure 5B). However, Snail did not affect the induction of properties of colorectal CSCs including CD44+ population growth, resistance to radiation, and ability of tumoursphere formation (Figure 5C,D), suggesting that the Snail-mediated EMT process might not be involved in SOX2-dependent induction of colorectal CSCs upon irradiation.

**Figure 6:**
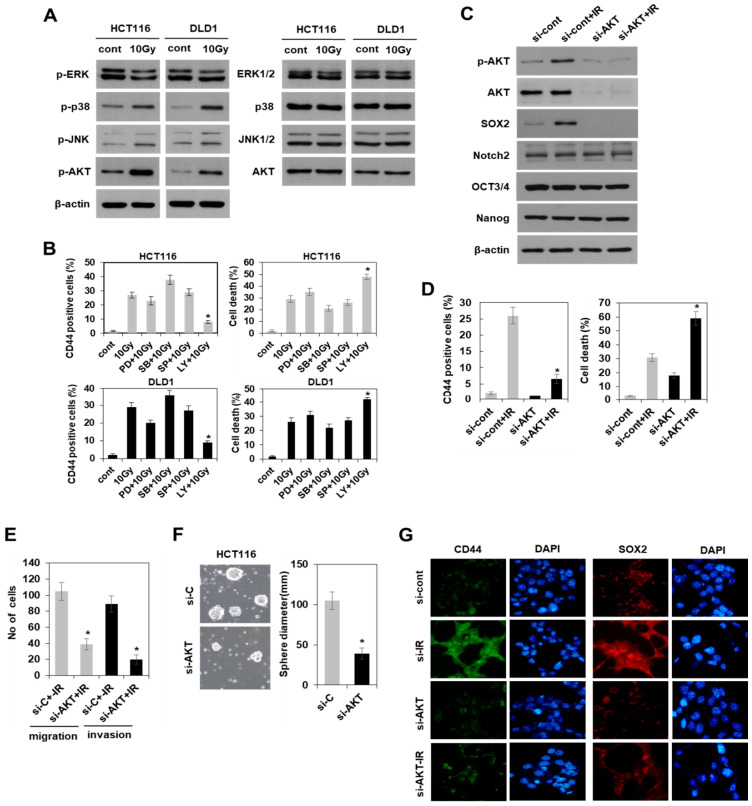
SOX2-dependent induction of colorectal CSCs was modulated by radiation-activated PI3K/AKT pathway, but not MAPK pathway. (**A**) Immunoblotting for mitogen-activated protein kinase (MAPK) pathway activation (p-ERK, ERK1/2, p-p38, p38, p-SAPK/JNK, JNK1/2) and phosphatidylinositol 3-kinase (PI3K)/AKT pathway activation (p-AKT, AKT) on day 2 after radiation (10 Gy) in radioresistant colorectal cancer cells (HCT116 and DLD1). (**B**) Analysis of CD44+ cell population by flow cytometry in HCT116 (left panel) and DLD1 (right panel) cells. The cells were exposed to radiation in the absence or presence of a pharmacological inhibitor of ERK pathway (PD98059, 10 μm), p38 pathway (SB203580, 10 μm), SAPK/JNK pathway (SP600125, 10 μm) and PI3K/AKT pathway (LY294002, 10 μm) for 2 days. Data are shown as mean ± SD (*n* = 3) with * *p* < 0.05 for the pairwise comparisons of CD44+ cell population between irradiated cells with inhibitors and the reference group (i.e., 10 Gy). (**C**) Immunoblotting for the expression of CSC-related proteins (SOX2, Notch2, OCT3/4, and Nanog) on day 2 after radiation (10 Gy) in AKT siRNA-transfected HCT116 cells. (**D**) Analysis of CD44+ cell population (left panel) and apoptotic cells (right panel) by flow cytometry in AKT siRNA-transfected HCT116 cells. Data are shown as mean ± SD (*n* = 3). * *p* < 0.05 compared si-Cont + IR to si-AKT + IR. (**E**) The migration and invasion on day 2 after irradiation (10 Gy) of the AKT siRNA-transfected HCT 116 cells were quantified. Bars indicate the measurements of migration and invasion. * *p* < 0.05 compared si-Cont + IR to si-AKT + IR. (**F**) Tumoursphere-formation assay was performed to evaluate self-renewal ability of CSCs in AKT siRNA-transfected cells. Data are shown as mean ± SD (*n* = 3). * *p* < 0.05 versus si-Cont. (**G**) Cells were stained with an anti-CD44 (green) and anti-SOX2 (red) antibody. Nuclei were counterstained with DAPI (blue). Si-Cont: control siRNA, si-AKT: AKT siRNA, CSCs: cancer stem-like cells.

Since SOX2 expression is important for inducing the characteristics of CSCs as observed in our study and from previous reports [14,15], we attempted to identify the upstream regulator of SOX2. To do so, we investigated the activation of mitogen-activated protein kinase (MAPK) and phosphatidylinositol 3-kinase (PI3K)/AKT pathways, known to regulate SOX2 under other conditions [26,27,28,29]. With western blotting, we found that radiation activated both MAPK and PI3K/AKT pathways except ERK activation (Figure 6A). Treatment with the pharmacological inhibitor of PI3K/AKT pathway (LY294002), but not the inhibitors of ERK (PD98059), p38 (SB203580), and SAPK/JNK (SP600125) pathways, dramatically suppressed radiation induced CD44 expression, which is a marker of colorectal CSCs and radioresistance in HCT116 and DLD1 cells (Figure 6B). The concentrations of the inhibitors used were referenced to previous studies, including our report [30]. Based on AKT silencing, we further confirmed the function of PI3K/AKT as an upstream regulator of SOX2-dependent induction of colorectal CSCs by observation of the reduced expression of SOX2 and CD44, as well as CSC properties, such as radioresistance, in vitro metastatic potential, and tumoursphere formation (Figure 6C–E). Moreover, immunocytochemistry further supported the AKT-dependent expression of both CD44 and SOX2 in irradiated HCT116 cells with or without AKT siRNA (Figure 6G). Collectively, these results suggested that radiation enhanced PI3K/AKT/SOX2 axis promoted the induction of colorectal CSCs in radioresistant colorectal cancer cells.

## 4. Discussion

The stemness program plays an important role in maintaining the properties of CSCs due to self-renewal, which is a hallmark of cancer-initiating cells. Recent studies have shown that SOX2 is aberrantly expressed and involved in the maintenance of properties of colorectal CSCs, including spheroid-like growth and metastatic potential [14,15]. Here, we extended these studies to demonstrate that SOX2 is regulated by the PI3K/AKT pathway and contributes to the induction of colorectal CSCs in response to radiation. By comparative analysis of radiation-induced population of CSCs in both radioresistant and radiosensitive colorectal cancer cells, we found that radioresistant cells such as HCT116 and DLD1 specifically increased the CD44+ population after irradiation, which is one of the properties of CSCs. Interestingly, we also found that the radiation-induced activation of PI3K/AKT pathway functions as an upstream regulator of SOX2-dependent induction of CSCs in colorectal cancer.

In this study, we report that radiation-enriched CD44+ cells exhibited colorectal CSC properties including resistance to radiation, enhanced in vitro metastatic potential, and a spheroid growth pattern. CD44 is a receptor of hyaluronan and is a transmembrane glycoprotein that participates in many cellular processes, including growth, survival, differentiation, and motility [31,32,33,34]. CD44 is considered a more selective marker of colorectal CSCs than CD133, because the properties of colorectal CSCs are not regulated by CD133 modulation [19,20,35,36]. Consistent with this, our comparative study between radioresistant and radiosensitive colorectal cancer cells showed that CD44 expression, but not that of CD133, was selectively increased in radioresistant colorectal cancer along with acquiring the properties of colorectal CSCs after irradiation. A recent study reported that SOX2 expression primarily coincided with CD44+ and ALDH1+ population in pancreatic CSCs [37] and CD44+ and CD24+ in colorectal cancer [14]. Indeed, we also observed that FACS-sorted CD44+ cells showed an upregulation of SOX2 expression and demonstrated its important role in modulating the CD44+ population growth and the properties of CSCs in colorectal cancer using both knockdown and overexpression of SOX2, which is consistent with previous reports. Notably, in our study, this functional relationship occurred in response to radiation, indicating that radiation affects SOX2-dependent induction of CD44+ population.

Factors that are important for self-renewal in stem cells are found to be dysregulated in human malignancies. SOX2 expression has been implicated in the control of colorectal CSC properties; however, the related signaling pathways are less understood. In a previous study, SOX2-induced CSCs in cervical and pancreatic cancer have been linked to epithelial-mesenchymal transition (EMT)-related factors [37,38]. However, these studies were considered controversial. Han et al. reported a role for SOX2 in EMT and increased in vitro metastatic potential, such as in migration and invasion in colorectal cancer [24], while Lundberg et al. reported that SOX2 mediated induction of CSC characteristics in an independent manner [14]. In our system, the ability of migration and invasion was dramatically regulated by SOX2-modulated Snail expression, known as a master regulator of EMT. However, we observed that Snail did not affect the induction of colorectal CSC properties, including CD44+ population growth, resistance to radiation, and the ability of tumoursphere formation. This suggested that the Snail-mediated EMT process might not be involved in SOX2-dependent induction of colorectal CSCs, although we could not exclude the possibility of involvement of other regulators of EMT process or factors related to tumor microenvironment [39] affected by irradiation. Therefore, it is likely that SOX2 modulates either EMT process or CSC induction through alternative pathways, at least in response to radiation. Further studies are required to clarify the relationship between EMT and CSCs induction.

An elucidation of the signaling pathways that govern the SOX2-dependent induction of CSCs is also required for devising an optimal targeted therapy. Considering that MAPK and PI3K/AKT pathways, in addition to being activated by radiation [40], were associated with resistance to therapy and tumorigenicity in cancer cells [41,42], we investigated the effect of the inhibitors of MAPK and PI3K/AKT for induction potential of CSCs. It was found that radiation activated the genes of both MAPK and PI3K/AKT pathways, consistent with previous reports, except ERK. Interestingly, the induction of SOX2 and CSC characteristics, including CD44+ cells in colorectal cancer, were only affected by the inactivation and downregulation of PI3K/AKT following irradiation. This finding is contradictory to the report by Wang et al., [43] who showed that activation of both AKT and MAPK pathways was involved in the induction of properties of colorectal CSCs, such as the colony formation ability in primary colon cancer cells. These differences can be explained using target cells with differential markers of CSCs and the response to stresses. To isolate colorectal CSCs, Wang et al. used CD133, a colorectal CSC marker, and characterized cells with or without CD133 expression under non-stress conditions for CSC properties, whereas we used CD44, which was specifically induced by radiation stress. Furthermore, the involvement of genes in the PI3K/AKT pathway in SOX2 regulation in breast and nasopharyngeal carcinoma has recently been reported [28,29]. Therefore, this is an interesting finding that radiation-activated PI3K/AKT pathway genes were essential for the SOX2-dependent induction of colorectal CSCs, and it is potentially an effective therapeutic target for CSCs in colorectal cancer activated by radiation.

## Data Availability

Not applicable.
